# USING LATENT CLASS GROWTH ANALYSIS TO IDENTIFY SUBGROUPS OF OLDER ADULTS WITH SHARED TRAJECTORIES OF FRAILTY STATUS

**DOI:** 10.1093/geroni/igae098.3222

**Published:** 2024-12-31

**Authors:** Hillary Spangler, Wenyi Xie, David Lynch, Feng-Chang Lin, John Batsis

**Affiliations:** University of North Carolina School of Medicine, Chapel Hill, North Carolina, United States; University of North Carolina, Chapel Hill, North Carolina, United States; University of North Carolina School of Medicine, Chapel Hill, North Carolina, United States; University of North Carolina, Chapel Hill, North Carolina, United States; University of North Carolina, Chapel Hill, North Carolina, United States

## Abstract

**Background:**

Frailty is a geriatric syndrome with significant clinical heterogeneity among older adults. Identifying groups experiencing different frailty trajectories is critical for identifying those at high risk of frailty progression and prevention.

**Methods:**

We used data from the National Health and Aging Trend Study (2011-2020), a cohort of Medicare beneficiaries >65 years. Individuals were categorized using Fried’s frailty phenotype. We included older adults (n=6,904) with ≥2 interviews with frailty status (improving, stable, worsening). We used latent class growth analysis (LCGA) to identify patterns of change in frailty status and the impact of social determinants of health (SDOH: transportation, family support, income, education) on frailty trajectories. We defined rural by The Office of Management and Budget’s standards.

**Results:**

Participants were 56.9% female, median age of 70-74. At baseline, 24.6% were robust, 50.4% were pre-frail, and 25.0% were frail. LCGA identified three trajectories over 10 years. Most participants (54.1%) had a stable trajectory, with almost no change in frailty status. Fewer participants (29.1%) had an improving trajectory, initially experiencing a decrease in the number of frailty items at ~6 years, followed by a slow increase over time. The smallest group (16.9%) had a worsening trajectory, with an increasing number of frailty components over time. The worsening trajectory group was older at baseline, majority female. Marital status, lower education and income levels, and Medicaid enrollment were associated with higher odds of worsening frailty trajectory.

**Conclusion:**

SDOH and rurality were associated with progressive frailty trajectories, highlighting the need for policy-level interventions for frailty mitigation.

